# Semi‐Quantitative Monitoring of Plant‐Arthropod Interactions by eDNA Metabarcoding of Individual Flowers and Leaves

**DOI:** 10.1002/ece3.73831

**Published:** 2026-06-30

**Authors:** Arndt Schmidt, Joanna Hank, Merve Tello, Michael Erik Grevé, Christian Ulrich Baden, Christian Maus, Henrik Krehenwinkel

**Affiliations:** ^1^ Department of Biogeography University of Trier Trier Germany; ^2^ Bayer AG Monheim am Rhein Germany

**Keywords:** agriculture, eDNA, metabarcoding, pollinator

## Abstract

Environmental DNA (eDNA) analysis has revolutionized our ability to study plant‐arthropod interaction diversity. However, the method has one main limitation: while it accurately recovers the presence of individual species, it has limited quantitative recording capabilities. Recent work suggests that this problem can be overcome by processing replicate eDNA samples at individual sites. The naive occupancy of a species within a replicate sample approximates its relative abundance or its interaction strength with the sampled matrix. However, this approach will lead to a significant increase in the necessary workload and costs, especially as the usually applied filtering of eDNA samples is laborious and expensive. Here, we explore the option to directly extract eDNA from individual leaves and flowers omitting a filtration step. We test different isolation protocols and compare the results with a conventional filtration‐based protocol. We show that simple precipitation suffices to recover community‐level eDNA of arthropods from individual flowers, significantly reducing costs and efforts. We then test our optimized protocol in two apple orchards in Germany, where we sample individual leaf and flower samples. Our protocol reliably recovers typical orchard‐associated arthropod species across broad ecological guilds. The naive occupancy‐based abundances of these species reflect the expected community composition in orchards very well. For comparative purposes, we also performed a conventional, quantitative bee monitoring in the orchards using window traps. Based on this monitoring, we show that replicate sampling of flowers is even a promising option to semi‐quantitatively recover bee pollinators and their ecological interaction diversity from eDNA. Simplified replicate eDNA sampling will considerably improve our ability to characterize arthropod communities.

## Graphical abstract

Terrestrial eDNA analysis is currently revolutionizing our ability to monitor arthropod plant interactions. But while the approach has been shown to accurately recover arthropod communities which interacted with a flower, its quantitative capabilities are limited. Here, we test using occupancy to quantify arthropod communities in apple orchards using single flowers and leaves, and test different extraction methods for those samples.

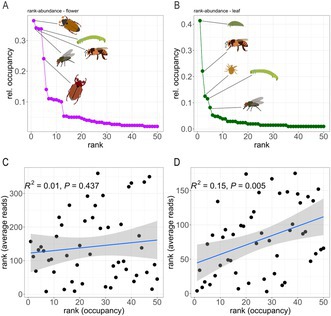

## 1 Introduction

There is a broad spectrum of use cases for innovative biodiversity monitoring methods in fundamental as well as applied research (Chintala et al. 2025; Stephenson 2020). To effectively monitor biodiversity, however, it is not only necessary to document the presence and absence of species, but also to record their abundances and the interactions between organisms and their environment (Thébault and Loreau 2005; Noss 1990; Windsor et al. 2023; Puig‐Gironès and Real 2022). Characterizing the interaction diversity in an ecosystem is highly important to understand its dynamics. However, the detection of biotic interactions is usually very time‐consuming, as it is based on visual observations for every interacting species pair (Bonelli et al. 2020; Gibson et al. 2011; Greenstone 1999; Nyffeler et al. 1994; Ono et al. 2008; Nielsen et al. 2011). In recent years, eDNA metabarcoding has greatly contributed to our ability to monitor interactions (Johnson et al. 2023; Schmidt et al. 2025; Thomsen and Sigsgaard 2019). The method is especially well‐suited to detect arthropod‐plant interactions. Simple eDNA wash‐offs from plant surfaces suffice to characterize an entire community of interacting arthropod species across all guilds interacting with the plant, including herbivores, pollinators, predators, and parasitoids (Stothut et al. 2024; Weber et al. 2024; Schmidt et al. 2025). This approach is considerably faster than conventional monitoring of plant‐arthropod interactions. By providing a fast and efficient way to detect interactions, eDNA promises to revolutionize the assessment of ecosystem diversity and dynamics (Thomsen and Sigsgaard 2019).

However, eDNA‐based biodiversity monitoring so far entails one important disadvantage: a quantitative assessment is hardly feasible up to now (Ruppert et al. 2019). As the method is based on PCR, amplification bias between different taxa in an arthropod community can lead to highly skewed abundance estimates (Elbrecht and Leese 2015; Fonseca 2018; Krehenwinkel et al. 2017). Different eDNA deposition rates between taxa can additionally skew the recovered species abundances (Friedrich et al. 2024). But species abundance distributions are a critical element of community diversity. Recent work suggests a simple solution to the problem: by collecting replicated eDNA samples at the same site, species can be classified into abundance classes. Abundant species will be present in all or most collected samples, while rare ones will only occur in one or a few (Bush et al. 2023). Commonly interacting arthropods will be found in most or every eDNA sample from a given plant species, while infrequently interacting ones will be found only in individual samples (Schmidt et al. 2025). Recent work shows that this approach enables semi‐quantitative monitoring of arthropod community diversity and arthropod‐plant interactions (Schmidt et al. 2025). Only about 20 eDNA samples already suffice to approximate abundance distributions of species in a community.

However, the necessity to collect replicate samples at each site or for individual plant species can lead to considerably increased cost and effort for eDNA‐based biomonitoring. Especially the filtration of eDNA from wash‐offs from plants and the DNA isolation step are relatively expensive, as every sample has to be washed, filtered, and isolated (Schmidt et al. 2025; Weber et al. 2024; Valentin et al. 2020). While it has already been shown that DNA extractions of single flowers work very well for recovering eDNA, we here try to further minimize the cost and workload, as most of the single flower extractions so far rely on extraction kits (Harper et al. 2023; Thomsen and Sigsgaard 2019; Johnson et al. 2023).

We use single leaves and single flowers for direct eDNA isolation, while the filtration step is entirely omitted. In addition, we make DNA isolation cheaper by miniaturizing and simplifying it. We hypothesize that (1) eDNA isolation can be simplified by direct isolation from individual leaves or flowers, enabling fast and high‐throughput generation of replicate samples, and (2) that this way, semi‐quantitative information can be generated at low cost and effort, allowing us to quantitatively monitor plant‐arthropod interactions, for example, pollinator specificity.

To test these hypotheses, we conducted several experiments. We first explored the possibility of simplifying DNA isolation protocols for recovering eDNA from individual cherry blossoms. These isolation protocols range from simple alcohol wash‐off omitting extraction entirely, to different DNA isolation protocols. In the second experiment, we test the potential of the optimized eDNA isolation protocol in apple orchards on individual flower and leaf samples to recover arthropod communities qualitatively and quantitatively with a focus on bee pollinators.

## 2 Materials and Methods

### 2.1 Experiment 1: Identifying the Optimal DNA Isolation Approach

#### 2.1.1 Field Collection and DNA Isolation

We conducted a preliminary survey to find a cheap and fast way of extracting eDNA from single flowers. Therefore, we collected single cherry blossoms on the campus of Trier University and placed them individually into pre‐labeled 2 mL tubes (Eppendorf, Hamburg, Germany) and froze them immediately after collecting. For extracting the eDNA, we tested four different methods:

1. Washing flowers and filtering the wash off (Fblo): This is the standard approach to recover arthropod eDNA from plants. Eight single blossoms were placed individually into zip‐lock bags, and 30 mL of sterile water was added. The samples were then shaken for one minute, and the water was transferred into a filter syringe (Poulten and Graf Wertheim, Wertheim, Germany) and filtered through a cellulose acetate filter with a pore size of 0.45 μL (Sartorius, Göttingen, Germany). The DNA from the filter was then extracted using the Blood and Tissue kit (Qiagen, Hilden, Germany) following the manufacturer's protocol. The extraction costs for one sample are roughly 10€ (June 2025).
2. Alcohol precipitation of eDNA (IsoG): This is the simplest and cost‐efficient DNA isolation approach. 600 μL of 2‐Propanol (Roth, Karlsruhe, Germany) and 1 μL of GlycoBlue (Invitrogen, Waltham, United States) were added individually to eight of the single cherry blossoms. The tubes were shaken for one minute, and afterwards the blossoms were removed using sterile forceps. After spinning the tubes down for 3 min at a speed of 14,000 rpm and removing the supernatant, 300 μL of 70% Ethanol was added. The tubes were centrifuged at 14000 rpm for one minute, and the supernatant was poured off. In the end, the DNA was hydrated. Extracting one sample using this method costs roughly 1€ (June 2025).
3. Isolation by lysis and precipitation (Pure): This approach is a standard DNA isolation protocol, involving cell lysis, purification, and DNA precipitation. The Puregene extraction kit (Qiagen, Hilden, Germany) was used for eight single cherry blossoms using a homemade lysis buffer (10 mM Tris pH 8, 100 mM NaCl, 10 mM EDTA pH 8, 0.5% SDS). The manufacturer's protocol was followed, with the incubation time adjusted to half an hour and the volumes multiplied by three. The costs to extract one sample are roughly 2€ (June 2025).
4. Spin column‐based extraction (Bloo): This approach is similar to the previous one. The difference is that a spin column‐based DNA isolation was used. Four remaining individual cherry blossoms in 2 mL tubes were used to test DNA extraction using the Blood and Tissue kit (Qiagen, Hilden, Germany) directly from the blossoms. The manufacturer's protocol was followed. The costs for one sample are roughly 6€ (June 2025).

All the different extraction methods were performed separately in a laboratory room dedicated to DNA isolation. In addition, negative controls for every isolation method were run along with the samples.

#### 2.1.2 PCR Amplification and Sequencing

For amplification, the primer combination fNoPlantF_270 (forward primer, RGCHTTYCCHCGWATAAAYAAYATAAG) and mlCOIintR_W (reverse primer, GRGGRTAWACWGTTCAWCCWGTNCC) was used, which targets the CO1 gene, identifying a broad range of arthropod taxa and does not amplify plant DNA. A 116 bp long fragment was amplified using the Qiagen Multiplex PCR kit (Qiagen, Hilden, Germany). PCR with 1 μL of each 5 μM primer, 5 μL of multiplex master mix, 2 μL of sterile water, and 1 μL of DNA was performed with 35 cycles and an annealing temperature of 46°C. The PCR was prepared under a PCR workstation (VWR, Radnor, USA) and performed in T100 Thermal Cyclers (Bio‐Rad, Hercules, USA). Gel images were used to check the success of the PCR. Afterwards, Illumina TruSeq Adapters (Illumina, California, USA) were attached to the 5'end using a dual index PCR with five cycles and an annealing temperature of 55°C. Gel images were again used to check the success of the index PCR, and an approximate volume of the products was pooled according to the intensity of the band. The pool was then cleaned using magnetic beads (1:1 sample to beads ratio, for recipe see (Jolivet and Foley 2015)). To check for contamination, a negative control was run along and sequenced for each isolation and PCR. Samples were sequenced using an Illumina MiSeq with a V2 kit (Illumina Inc., San Diego, USA).

### 2.2 Experiment II: A Field Test on eDNA Isolation From Individual Flowers and Leaves

#### 2.2.1 Field Collection and DNA Isolation

Apple orchards in two regions in western Germany were chosen as sampling locations. The first orchard is located in Heidelberg‐Kirchheim (Baden‐Wuerttemberg, Germany) at an altitude of 105 m above sea level and covers an area of approximately three hectares. On the fruit‐growing site, different varieties of apple, pear, and some other fruits are grown. The orchard is surrounded by agricultural land, fields, and an equestrian facility. Furthermore, in immediate proximity to the sampling area residues of green cuttings, e.g., wood and roots are stored. The second apple orchard is located in Burscheid (North Rhine‐Westphalia, Germany) and is approximately four hectares in size, at an altitude of 207 m. Different apple and pear varieties are grown in the sampling area, which is surrounded by agricultural land, a grassland area, and a small forest. Western honeybees ( *Apis mellifera* ) are kept at each sampling site.

To provide background information on the present bee community, two air eclectors were hung between apple trees at each sampling site, approximately 1.60 m above the ground, close to each other (~4 m distance). The air eclectors did not have any luring effect (e.g., yellow or white stripes) in order to catch only bees flying between the trees. The traps were emptied on every eDNA sampling date (Table S1).

Single flowers and leaves of the apple trees were collected for eDNA sampling. For the flowers, five trees were selected in every sampling area close to the two air eclectors, and on every sampling, ten flowers were picked from each of the five trees and placed individually in 2 mL pre‐labeled tubes. Flower sampling took place on three dates (Table S1), and the same five trees were sampled on each sampling date leading to 150 single apple flower samples for each apple orchard in total. Sampling began when the apple trees began to blossom. The second sampling took place in the midst of the blossoming period, and the last at the end (Table S1). Attempts were made to keep the sampling interval equal between the different sites, but due to rainy weather some sampling dates were shifted. For the leaf sampling, 15 single leaves, which had signs of feeding, were collected from trees close to the air eclectors and placed individually into 2 mL tubes on every sampling date. Leaf sampling started at the second flower sampling and was continued every two weeks after the last flower sampling. In total, Heidelberg was sampled ten times and Burscheid five times leading to 150 single leaf samples in Heidelberg and 75 in Burscheid.

To avoid cross‐contamination, single‐use latex gloves were worn and changed after every single sample, e.g., after every single apple blossom collected. The samples were cooled after they were taken and immediately frozen at −20°C on arrival at the laboratory until further processing.

Due to warm and rainy weather in spring 2024 in Germany, the apple blossom started earlier than expected. Heidelberg in particular experienced high temperatures, reaching 22°C in early April. The wet spring made eDNA sampling challenging, with the best‐case scenario being no rain before sampling, which could wash off the eDNA. As a result, some sampling dates had to be rescheduled, and some sampling days still had a bit of rain the day before sampling.

To prevent cross‐contamination, the entire working area of the laboratory room dedicated to DNA isolation was cleaned before the isolation process. In addition, negative controls were run for every DNA extraction batch. Since the Qiagen Puregene extraction protocol (Qiagen, Hilden, Germany) performed the best in the pre‐trial, as it had high values for ESV richness and read sums while also being relatively cheap and fast, for the extraction of eDNA from single flowers, eDNA isolation of the apple blossoms and apple leaves was done using this extraction protocol as described above (see 3, Isolation by lysis and precipitation).

Bee specimens caught in the air electors were processed separately from eDNA samples to avoid cross‐contamination. They were first sorted into honeybees ( *Apis mellifera* ) and wild bees. One leg of these unidentified bees was then collected, placed individually into 2 mL tubes, and DNA was isolated using the Puregene Extraction protocol (Qiagen, Hilden, Germany) in order to identify the species using metabarcoding.

PCR and sequencing were performed as described above. PCRs were run in duplicates. PCR and sequencing for eDNA and whole specimen samples were performed separately to prevent cross contamination. Again, negative controls for the PCRs were performed.

#### 2.2.2 Data Processing and Statistical Analysis

The data was analyzed using the APSCALE pipeline v2.1.1 (Buchner et al. 2022) with default settings. This pipeline is based on VSEARCH (Rognes et al. 2016) and cutadapt (Martin 2011). The exact sequence variants (hereafter ESV) were taxonomically assigned using the local blastn function in APSCALE, based on blast + (Camacho et al. 2009). The reference database used was Midori2 (Leray et al. 2022), and the thresholds for taxonomic assignment were as follows: 98%: species level, 95%: genus level, 90%: family level, 85%: order level, and < 85%: class level. In addition, only DNA fragments ranging from 60 bp–65 bp were retained. ESVs belonging to *Trombidiformes*, *Mesostigmata*, and *Sarcoptiformes* were merged into Acari.

Only ESVs present in both PCR duplicates were kept and summed up. To account for potential contamination, the reads for each ESV were summed up for the negative controls, and then the sum was subtracted from the corresponding ESVs for each sample. Only ESVs assigned to the phylum Arthropoda (without crustaceans) were retained, and reads below the threshold of four were set to zero to remove index carryover between samples.

For the statistical analysis, R 4.2.2 (R Core Team 2024) and RStudio 2024.12.0 (RStudio 2020) were used. They were extended with the R package vegan v2.6–4 (Oksanen et al. 2001) and tidyverse v 2.0.0 (Wickham et al. 2019). For plotting, ggplot2 v3.4.1 (Wickham 2016) and ggpmisc v0.5.2 (Aphalo 2025) were used. ESV richness and binary Jaccard dissimilarity were used as measures of α‐ and β‐diversity. To test for differences in the mean ESV‐richness and mean species richness, the pairwise Wilcoxon signed‐rank test with Bonferroni correction or the Wilcoxon test was used when the data were not normally distributed, and the *t*‐test when the data were normally distributed. The Shapiro–Wilk‐test was used to test for normal distribution. To test for differences in the order or genus composition, Fisher's exact test with a simulated *p*‐value was used.

We calculated the average reads for every species by dividing the sum of the reads of every species by the number of samples in which they were found. To explore the possibility of a quantitative assessment of the bee community, we first explored whether the read count reflects true abundances. For this purpose, we compared the true abundance of bees of each species as sampled in the traps over the entire sampling period with the total number of reads for each species recovered from the individual leaf and flower samples. In addition, we tested if using naive occupancy for eDNA samples better reflects the true abundance structure of the bee community. Naive occupancy is defined as the number of eDNA samples in which a bee species is present. This data was then compared to the true abundances of the bee species as sampled in the traps. In addition, we also explored if it is especially abundant bee species with a high naive occupancy that are associated with apple trees. Therefore, ecological information about these species was collected from literature (Westrich 2019).

## 3 Results

### 3.1 Experiment 1: Identifying the Optimal DNA Isolation Approach

**FIGURE 1:**
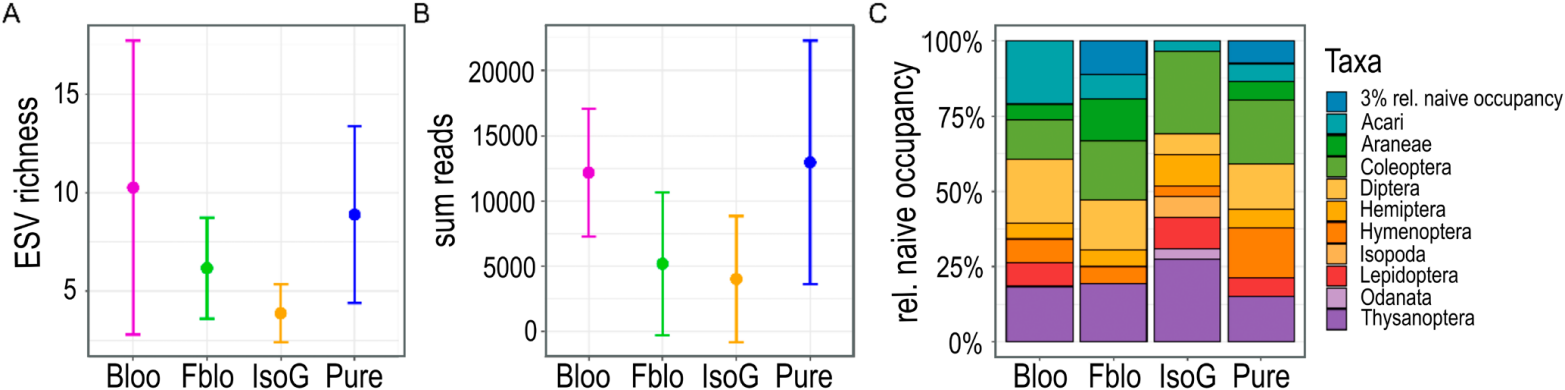
Recovery of cherry blossom‐associated arthropod communities with different extraction methods, which were Bloo (Spin column‐based extraction), Fblo (Washing flowers and filtering the wash off), IsoG (Alcohol precipitation of eDNA), and Pure (Isolation by lysis and precipitation). (A) Error bar plot of the recovered exact sequence variants (ESV) richness. (B) Read abundances for the ESVs recovered by the different methods. (C) Bar plot of order‐level taxonomic composition.

Diverse arthropod communities were recovered from the cherry blossoms; a total of 497,806 raw reads were obtained after sequencing, of which 215,739 reads were used for further analysis after filtering, consisting of 140 ESVs in 56 families and 18 orders. Arthropods from various trophic levels were represented by the recovered taxa, including herbivores, pollinators, detritivores, parasitoids, and predators. To find a fast and cheap protocol for extracting DNA from single blossoms, four different methods were tested. None of the approaches recovered significantly more ESVs when compared to each other (mean ESV richness: 6.2 ± 2.5 (Fblo), 3.9 ± 1.5 (IsoG), 8.9 ± 4.5 (Pure), 10.3 ± 7.5 (Bloo); Figure 1A, pairwise *t*‐test, *p* > 0.05). Pure had the highest recovery of total ESVs (53) followed by Fblo (31), Bloo (31), and IsoG (22). When comparing the total reads between the methods, there was also no significant difference (5193.7 ± 5470.5 (Fblo), 4026.9 ± 4830.1 (IsoG), 12960.8 ± 9314.2 (Pure), 12,169 ± 4872.9 (Bloo); Figure 1B, pairwise Wilcox‐test, *p* > 0.05). The order composition recovered by the different methods, however, was significantly different (Figure 1C, Fisher's exact test for all comparisons, *p* < 0.05). The four most frequently detected orders for the Fblo method were Coleoptera (16%), Diptera (16%), Thysanoptera (16%) and Araneae (14%), for IsoG Coleoptera (27%), Thysanoptera (27%), Hemiptera (10%) and Lepidoptera (10%), for Pure Coleoptera (20%), Hymenoptera (16%), Diptera (14%) and Thysanoptera (14%), and for Bloo, Acari (16%), Diptera (21%), Thysanoptera (18%) and Coleoptera (13%).

### 3.2 Experiment II: A Field Test on eDNA Isolation From Individual Flowers and Leaves

#### 3.2.1 Comparison of Arthropod Communities From Different Sample Types and Locations

**FIGURE 2:**
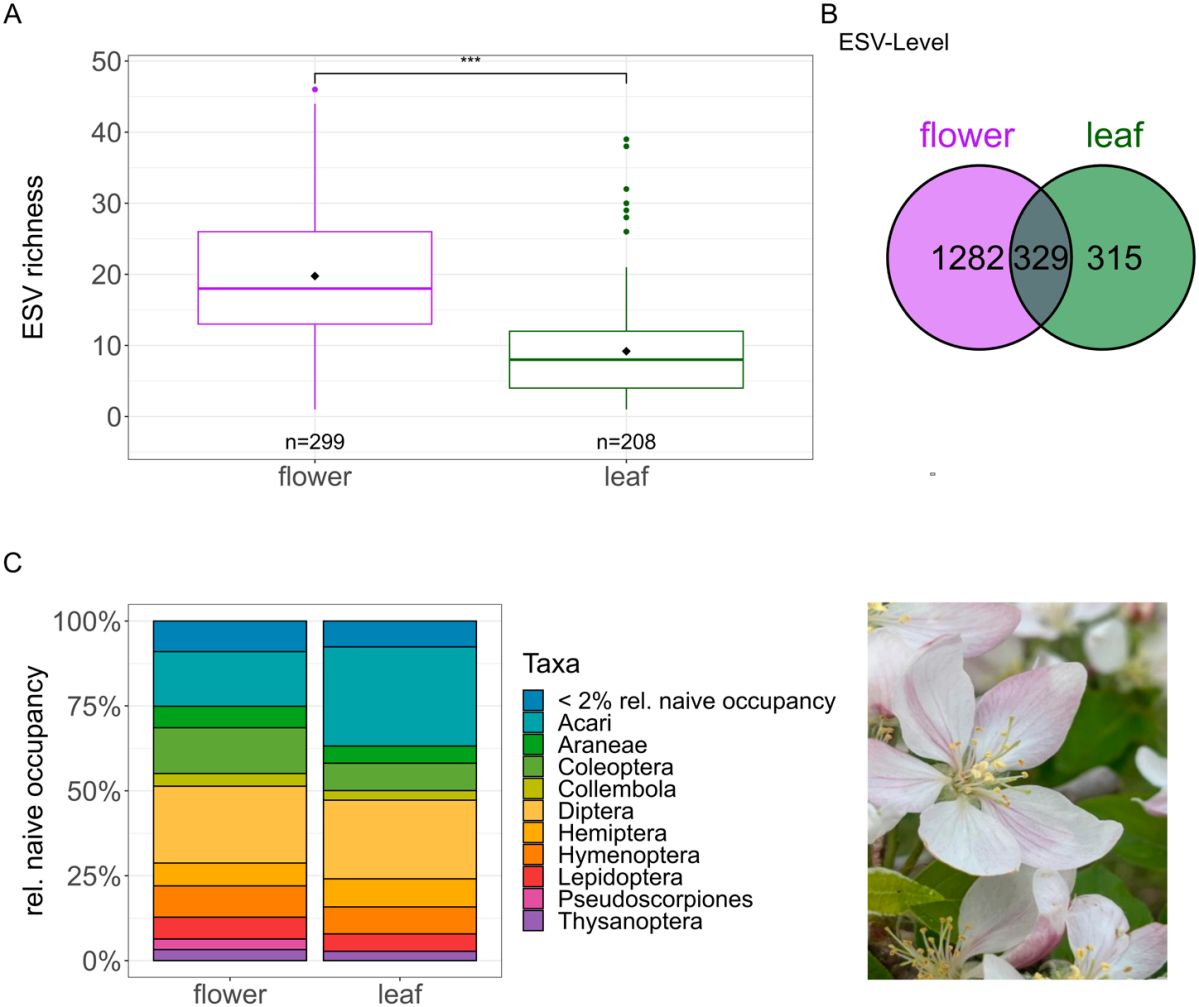
Arthropod communities associated with apple trees recovered from individual apple leaves and flowers (see photo). (A) Boxplot of the recovered exact sequence variants (ESV) richness. (B) Venn diagram of overlapping ESVs. (C) Bar plot of the order‐level taxonomic composition.

After sequencing, 23,375,002 raw reads were obtained, and after filtering 6,959,554 reads were used for further analysis with an average of 13,726.93 ± 12,158.24 reads for each sample, resulting in 1926 ESVs in 327 families and 46 orders. Various trophic levels were represented by the ESVs, including herbivores, pollinators, detritivores, parasitoids, and predators. To test whether it is possible to recover a sufficient amount of eDNA from individual leaves and flowers, we collected them individually in apple orchards. Samples with zero detected ESVs were excluded from the dataset (One flower, 17 leaves). Significantly more ESVs were recovered on average from the individual flowers compared to the individual leaves (Figure 2A; mean ESV richness 19.76 ± 9.66 (flower) and 9.18 ± 6.66 (leaf), Kruskal‐Wallis test: *p* < 0.05). Furthermore, the total recovered ESV number from the individual flowers was higher than that from the individual leaves (Figure 2B; 1611 vs. 644 ESVs). Only a small proportion of the ESVs were detected in both tissues (329 ESVs, 17.1%). The biggest proportion of ESVs with 66.6% of all ESVS was unique to the flower samples (1282 ESVs), while only 16.4% of the ESVs were unique to the leaf samples (315 ESVs). Despite the big difference in total ESVs, the order composition recovered by the two tissues was similar (Figure 2C; Fisher's exact test, *p* = 0.67). The three most frequently detected orders made up nearly 50% of all detections and comprise Diptera (23% flower and leaf), Acari (16% flower, 29% leaf), and Coleoptera (13% flower, 8% leaf).

#### 3.2.2 Quantification of the Recovered Plant‐Associated Arthropod Communities

**FIGURE 3:**
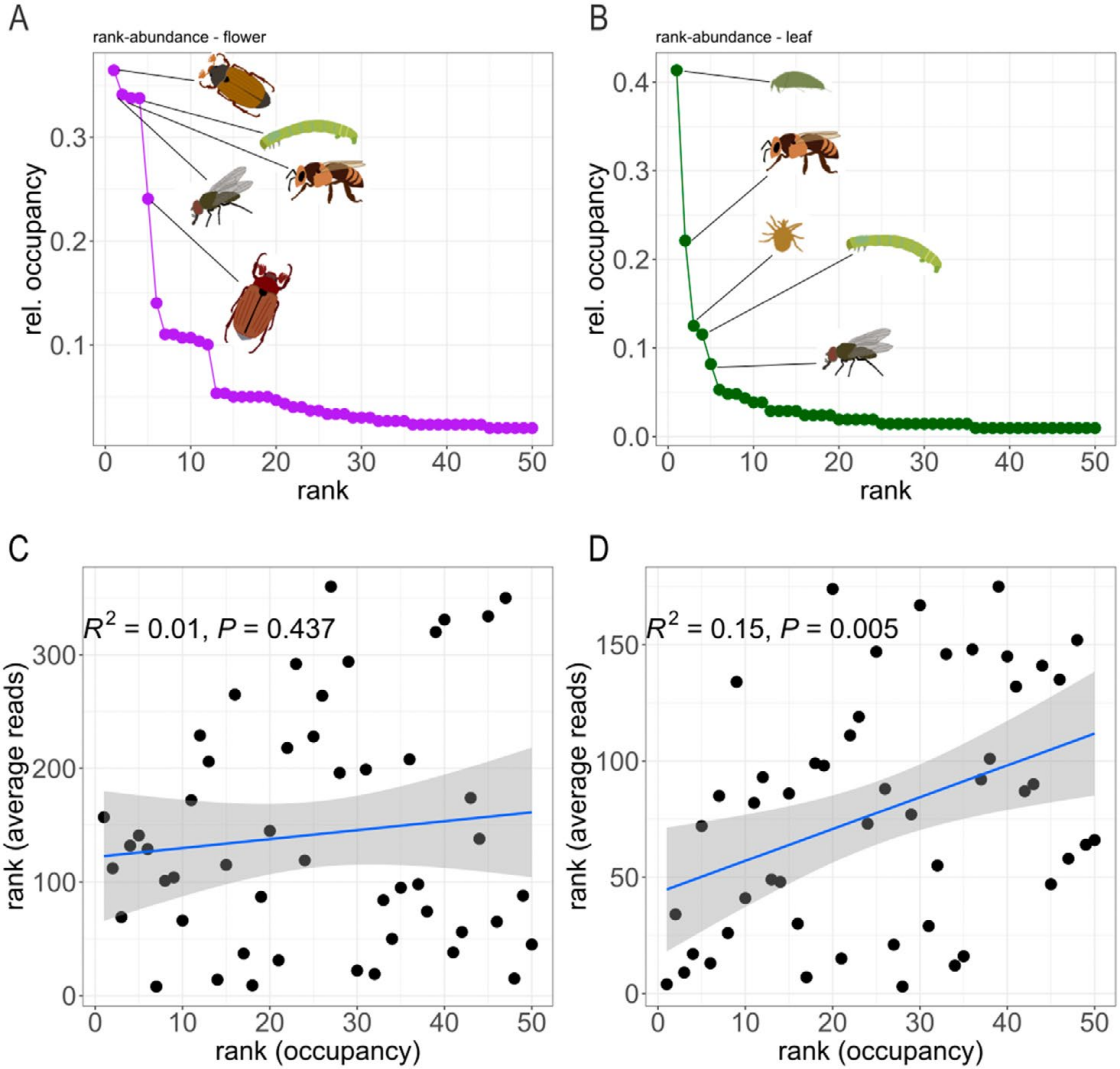
Rank‐abundance plot for the 50 species with the highest naive occupancy for (A) apple flowers and (B) apple leaves. Comparison of the 50 species with the highest naive occupancy against the rank they have for the average reads for flower (C) and leaf (D). For the detailed species list, Table S2 (Pictograms made by Lina Frank).

To explore the use of naive occupancy as a proxy for abundance, we also calculated rank‐abundance plots using naive occupancy as abundance. In this case, naive occupancy was defined as the number of samples in which a specific species was found. In total, we analyzed 299 individual apple flowers and 208 individual apple leaves. Some species were found in many samples, but most of the species were only found a few times (Table S2). Five species were detected very frequently on the apple blossoms (Figure 3A). These are *Melolontha melolontha* (rank: 1; naive occupancy: 109), *Delia platura* (rank: 2; 102), *Apis mellifera* (rank: 3; 101), *Operophtera brumata* (rank: 4; 101), and *Melolontha hippocastani* (rank: 5; 72). *Aculus schlechtendali* was found in roughly 40% of all individual leaf samples (rank 1; 86). Also, *Apis mellifera* (rank 2; 46), *Typhlodromus pyri* (rank 3; 26), *Operophtera brumata* (rank 4; 24), *Delia platura* (rank 5; 17) and *Dysaphis plantaginea* (rank 6; 11) were detected frequently on the individual leaves (Figure 3B). In addition, we compared the rank of the 50 species with the highest naive occupancy against the rank of those species for average reads. For the flower samples there is no significant correlation (Figure 3C) while the results from the leaf samples have a significant correlation, which is, however, only very small (R^2^ = 0.15, Figure 3D).

#### 3.2.3 Bee Pollinator Recovery by the Different Sampling Types

**FIGURE 4:**
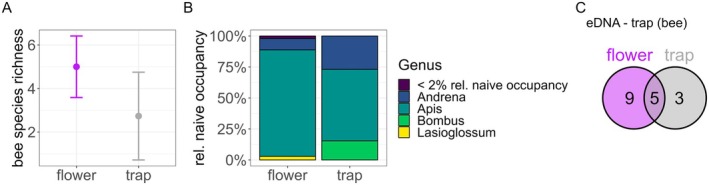
Recovery of bee pollinator communities from single apple flowers and bee species caught in traps in the apple orchards. (A) Bee species richness for the eDNA methods when merging every sample for every sampling day compared to the bee species richness recovered by the traps. (B) Bar plot of genus‐level composition of the bee community recovered by the two approaches. (C) Venn diagram of the recovered bee species counts for each method and the overlap.

In order to test whether it is possible to use individual leaves and flowers from apple trees to detect bee pollinators, these two methods were compared to data collected from window flight traps. Here, we only compare the single apple flowers and the bees caught during the period of flower sampling. After filtering, 40,691 reads were left representing a total of 14 bee species, while 8 different bee species were sampled in the traps. To make the data more comparable to the trap data, all samples for every sampling type and date were merged. The average number of bee species recovered from the individual flowers (5 ± 1.41 bee species) was significantly higher compared to the ones caught in the traps (2.73 ± 2 bee species; Figure 4A; Wilcoxon‐test, *p* < 0.05). The overlap between the 14 bee species detected by eDNA and the eight caught in the traps is roughly 30% (5 bee species), leading to nine bee species exclusively detected by the flower eDNA samples and three in the traps (Figure 4C). Most of the bee species found by only one method were found only a few times by that method (Figure S1 A,B). The bee genus composition between the two sampling types was significantly different (Figure 4B; all Fisher's exact tests, *p* < 0.05). The genus *Apis* was extremely dominant in all sampling methods, reaching 86% (flower) and 58% (trap) of detections (Figure 4B). *Andrena* was also detected frequently, with 27% (trap) and 9% (flower). Species from the genus *Bombus* were only detected in traps (15%), whereas *Lasioglossum* species were only found in flower samples (3%) (Figure 4B).

#### 3.2.4 Quantification of the Recovered Plant‐Associated Bee Communities Using Naive Occupancy

**FIGURE 5:**
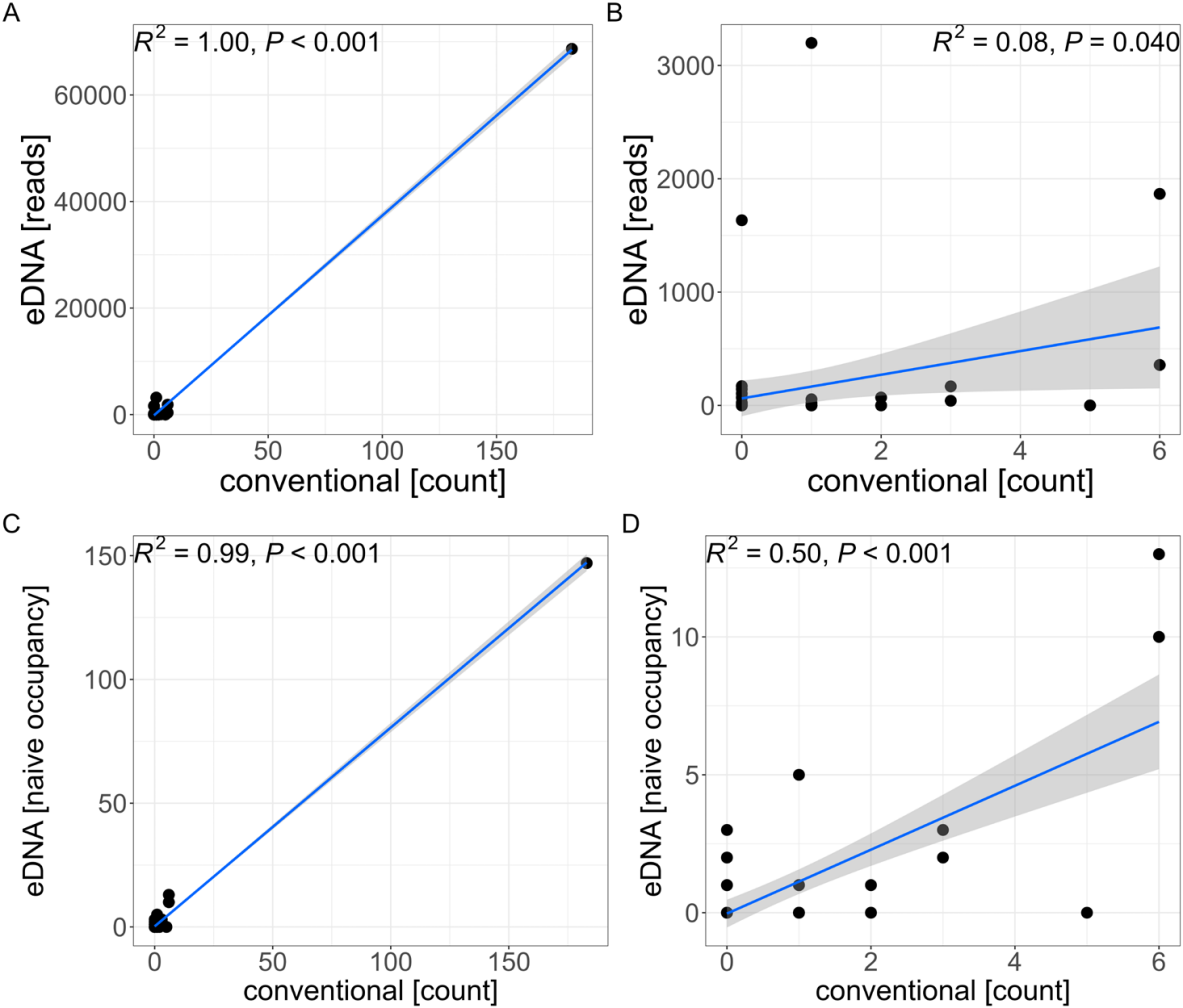
Quantitative recovery of bee communities using eDNA recovered from individual apple leaves and flowers. Gray areas represent the standard deviation. (A) Scatterplot of true abundances of bee species detected by the traps on the x‐axis and eDNA read counts for the same species on the y‐axis with (A) and (B) without Apis mellifera. (C) Scatterplot of true abundances of bee species detected by the traps on the x‐axis and naive occupancy detected by eDNA for the same species on the y‐axis with (C) and (D) without Apis mellifera.

A significant correlation between read abundances in eDNA (flower and leaf) and bee counts from the traps was found (Figure 5A, R^2^ = 1.00, *p* < 0,001). However, the highly abundant *Apis mellifera* was responsible for the strong correlation. When *Apis mellifera* was removed from the dataset, the correlation of abundances from the traps and read abundances from the eDNA analysis was only weak (Figure 5B, R^2^ = 0.08, *p* = 0.033). In contrast, naive occupancy correlates very well with the number of bees found in the traps, both when *Apis mellifera* is included (Figure 5C, R^2^ = 0.99, *p* < 0.001), as well as when it is excluded (Figure 5D, R^2^ = 0.5, *p* < 0.001). Naive occupancy was defined as in how many eDNA samples in which the bee species was found.

## 4 Discussion

### 4.1 Simplifying eDNA Isolation for High Throughput Replicate Sampling of Plant‐Arthropod Interactions

An especially laborious and costly step in an eDNA analysis protocol is the filtering of samples and DNA isolation from the filter. This precludes the routine use of replicate sampling in current eDNA studies (Schmidt et al. 2025; Weber et al. 2024; Valentin et al. 2020). Avoiding the need for expensive filtration and instead performing a direct DNA extraction will significantly reduce this cost and effort. It should be noted that the cost reduction is only achieved for DNA isolation, not for follow‐up steps like PCR and sequencing. Filtration and DNA isolation, however, are the most expensive parts of most eDNA protocols. Our first experiment shows that this is indeed a viable option. All of the extraction methods tested by us are potentially suited for eDNA analysis from individual flowers. As expected, conventional filtering approaches performed well and recovered a diverse arthropod community, which is expected on early flowering cherry trees in Germany with species like *Taeniothrips inconsequens* , *Epurea melanocephalia*, and *Apis mellifera* . The cheapest and simplest method for DNA isolation was alcohol precipitation. This approach, however, showed a reduced recovery of ESVs and arthropod reads. A very good compromise between labor, cost, and taxon recovery was found by performing a precipitation‐based DNA extraction directly on a flower sample (Thomsen and Sigsgaard 2019). We used a simple kit for this purpose, but any other and cheaper extraction protocol will probably suffice here. We thus used this approach for all follow‐up experiments and also recommend it for eDNA isolation from individual flowers or leaves. However, this approach has limits as large flowers do not fit into the small tubes. Adjustments are needed for the protocol when dealing with larger flowers, e.g., using larger volumes of buffers. Furthermore, using multiple flowers in one tube can increase the detected diversity, for which buffer volumes have to be increased (Thomsen and Sigsgaard 2019).

Using this approach in an apple orchard, we show its excellent performance in field settings. We recovered typical apple tree‐associated arthropod species from various orders and spanning diverse trophic ecologies, such as typical herbivore pests (*Aculus schlechtendali*, *Operophtera brumata* , *Dysaphis plantaginea*), predators (*Typhlodromus pyri*), and pollinators ( *Apis mellifera* ), showing the potential of the method to monitor orchards on different trophic levels. We also found pronounced differences in the recovered communities from flowers and leaves, underlining these two plant compartments as distinct habitats for different interacting arthropods. For example, bee pollinators recovered a significantly higher richness on flowers than on leaves. The mentioned differences were even found between leaves and flowers from the same trees, reflecting the fine‐scaled community differentiation, which terrestrial eDNA analysis allows for detecting. Overall, these findings confirm our first hypothesis that direct eDNA isolation from individual flowers is feasible.

### 4.2 Quantifying Communities and Interaction Diversity by Replicate eDNA Sampling

So far, a main limitation of eDNA analyses is their ability for quantitative assessments (Ruppert et al. 2019). Due to numerous biases, eDNA read abundances in a community sample do frequently not reflect true species abundances (Elbrecht and Leese 2015; Fonseca 2018; Krehenwinkel et al. 2017). Recent work suggests that replicate sampling may be an excellent option to overcome this issue and generate abundance classes for species in a community (Bush et al. 2023). Alternatively, replicate samples can also be used to detect the interaction strength between species, for example, a pollinator and a flower (Schmidt et al. 2025) or a predator and its prey (Eitzinger et al. 2019). Our work shows that this is indeed feasible. By using simplified extraction protocols, replicate samples can be generated in large numbers for relatively little cost and effort. The rank abundance curves we generated are ecologically meaningful as they reflect abundance distributions expected in apple orchards very well. For example, *Apis mellifera* was detected in many samples, which is very much expected since colonies of this species are kept in every sampling site. Furthermore, apple tree‐associated arthropod species like *Operophtera brumata* , a known pest of the trees, *Dysaphis plantagerinea*, or *Aculus schlechtendali*, which are expected to be abundant in apple orchards, are frequently detected (Dib et al. 2010; Duso et al. 2010; Hand et al. 1987). The high naive occupancy of cockchafers (genus *Melolontha*) in Heidelberg can also be explained by an outbreak of this species when the sampling took place. In addition, we found that the naive occupancy of *Dysaphis plantagerinea*, an aphid, positively correlates with its predator *Coccinella septempunctata*. Moreover, there is no clear correlation between the rank calculated by naive occupancy and average read counts, indicating that primer amplification biases are reduced using this method. These findings suggest that community‐level composition information can be recovered by replicate eDNA sampling. This opens the option to monitor agricultural land for abundant pest species. For future work, it may be recommendable to create true rank abundance curves and compare them to the eDNA‐based ones we show here. This, however, requires considerable workload, as multiple arthropod sampling approaches have to be employed, and each collected specimen needs to be identified.

The approach also seems to work very well to detect and quantify the specificity of plant‐arthropod interactions. An arthropod that is specialized on a certain plant species will be detected on that plant in high naive occupancy. This is nicely shown by many specialized herbivores of apple trees being found in high naive occupancy in our data. But this also appears to work with pollinator communities, as we exemplarily show with bees. Pollinators will most likely only release a small amount of eDNA in comparison to other arthropods in their brief interactions with flowers, making them especially hard to detect. Recent work has already shown that bee communities can be quantified using replicate sampling of large bulk flower samples (Schmidt et al. 2025). Here, we show that this also works with samples as small as individual flower heads. Replicate sampling of flower heads approximates the relative abundances of different bee pollinators in a community. As expected, at all sampling sites, the western honeybee ( *Apis mellifera* ) is dominant in the traps and in the eDNA samples, again showing the potential of eDNA metabarcoding. But even when *Apis mellifera* is removed from the dataset, naive occupancy still yields good approximations of the abundances of other wild bee species.

In addition, when only comparing flower eDNA and the traps, as pollinators are more likely to interact with the flower than with the leaves, we were able to detect 60% of the bee species caught in the traps using flower‐derived eDNA in the flower sampling period, showing the potential of eDNA for detecting bee communities. eDNA gives us even more information about the bee community present in the apple orchard compared to the traps as we detect almost twice as many bee species as were caught in the traps.

Overall, our results also confirm our second hypothesis. Using replicate samples from individual flowers, with a simplified extraction protocol, semi‐quantitative information can be generated at low cost and effort. This allows us to quantitatively monitor plant‐arthropod interactions, for example, pollinator specificity in high throughput. This promises to greatly improve the ecological information delivered by arthropod monitoring. It should be noted that the occupancy used here does not provide true abundance information. Different species can only be grouped into abundance classes or classes of interaction strength. This is, however, an important step forward from a pure presence‐absence analysis in eDNA.

## 5 Conclusions

There is a strong need for efficient future arthropod monitoring, which incorporates information on species abundances and their ecological interaction diversity. This can be achieved by replicating the sampling of plant material and directly isolating eDNA from it. Direct DNA isolation provides a simple means to achieve high‐throughput replicate samples for future eDNA monitoring of plant arthropod interactions. Our results will thus contribute to a significant improvement of future arthropod monitoring programs.

## Author Contributions

**Arndt Schmidt:** conceptualization (equal), formal analysis (equal), investigation (equal), methodology (equal), visualization (equal), writing – original draft (equal). **Joanna Hank:** formal analysis (equal), investigation (equal). **Merve Tello:** formal analysis (equal), investigation (equal). **Michael Erik Grevé:** conceptualization (equal), methodology (equal), project administration (equal), resources (equal), supervision (equal), writing – review and editing (equal). **Christian Ulrich Baden:** conceptualization (equal), methodology (equal), project administration (equal), resources (equal), supervision (equal), writing – review and editing (equal). **Christian Maus:** conceptualization (equal), methodology (equal), project administration (equal), resources (equal), supervision (equal), writing – review and editing (equal). **Henrik Krehenwinkel:** conceptualization (equal), investigation (equal), methodology (equal), project administration (equal), resources (equal), supervision (equal), writing – original draft (equal).

## Funding

This work was funded by Bayer AG.

## Conflicts of Interest

The authors declare no conflicts of interest.

## Supporting information


**Table S1:** Sampling dates for the different sampling methods and the dates where the traps were emptied.
**Table S2:** Species list for the 50 most abundant species in the flower eDNA samples and leaf eDNA samples.
**Figure S1:** Comparison of the bee species detected by the conventional method and the eDNA methods (apple leaves and flowers). (A) Read abundance of the 5 bee species detected by both methods (eDNA and traps) and the 11 bee species detected exclusively by the eDNA method. (B) Relative abundance of the 3 bee species detected exclusively in the traps and the 5 bee species detected by the two methods.

## Data Availability

The data that support the findings of this study are openly available in datadryad at http://doi.org/10.5061/dryad.47d7wm3t0.
