# Supplementary material for: Semi‐Quantitative Monitoring of Plant‐Arthropod Interactions by eDNA Metabarcoding of Individual Flowers and Leaves
Source: Ecol Evol. 2026 Jun 30;16(7):e73831. doi: 10.1002/ece3.73831 (PMC13316126; doi:10.1002/ece3.73831)
Supplement: Supplementary file 1 — Table S1: Sampling dates for the different sampling methods and the dates where the traps were emptied. Table S2: Species list for the 50 most abundant species in the flower eDNA samples and leaf eDNA samples. Figure S1: Comparison of the bee species detected by the conventional method and the eDNA methods (apple leaves and flowers). (A) Read abundance of the 5 bee species detected by both methods (eDNA and traps) and the 11 bee species detected exclusively by the eDNA method. (B) Relative abundance of the 3 bee species detected exclusively in the traps and the 5 bee species detected by the two methods. [file ECE3-16-e73831-s001.docx]

Supplementary figures

Supplementary table 1: Sampling dates for the different sampling methods and the dates where the traps were emptied.


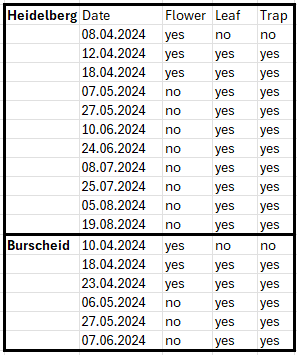


Supplementary table 2: Species list for the 50 most abundant species in the flower eDNA samples and leaf eDNA samples.

| **flower** |  |  |  |  |
| --- | --- | --- | --- | --- |
| Rank | Species | Occupancy | ecology | associated with apple tree |
| 1 | Melolontha melolontha | 109 | herbivore | Observed to occur (Lakatos and Tóth 2006) |
| 2 | Delia platura | 102 | herbivore/pollinator | Observed to occur (Boucher et al. 2021) |
| 3 | Apis mellifera | 101 | pollinator | Associ. closely (Verma and Dulta 1986) |
| 4 | Operophtera brumata | 101 | herbivore/pollinator | Associ. closely (Hand et al. 1987) |
| 5 | Melolontha hippocastani | 72 | herbivore | Observed to occur (Kula 2022) |
| 6 | Porcellionides pruinosus | 42 | detritivore | Not mentioned as associ. |
| 7 | Aculus schlechtendali | 33 | herbivore | Associ. closely (Ellis WN 2025) |
| 8 | Scathophaga stercoraria | 33 | pollinator | Not mentioned as associ. |
| 9 | Orthonychiurus folsomi | 32 | detritivore | Not mentioned as associ. |
| 10 | Scatopsciara atomaria | 32 | herbivore/pollinator | Not mentioned as associ. |
| 11 | Ommatoiulus sabulosus | 31 | detritivore | Not mentioned as associ. |
| 12 | Cylindroiulus caeruleocinctus | 30 | detritivore | Not mentioned as associ. |
| 13 | Armadillidium vulgare | 16 | detritivore | Not mentioned as associ. |
| 14 | Thrips major | 16 | herbivore | Observed to occur (Pobożniak 2008) |
| 15 | Aphodius granarius | 15 | detritivore | Not mentioned as associ. |
| 16 | Calliphora vomitoria | 15 | pollinator | Not mentioned as associ. |
| 17 | Lasius psammophilus | 15 | other | Not mentioned as associ. |
| 18 | Taeniothrips inconsequens | 15 | herbivore | Observed to occur (David A. J. Teulon et al. 1994) |
| 19 | Xenylla welchi | 15 | detritivore | Not mentioned as associ. |
| 20 | Epuraea melanocephala | 14 | herbivore/pollinator | Not mentioned as associ. |
| 21 | Lycoriella sativae | 13 | other | Not mentioned as associ. |
| 22 | Harmonia axyridis | 12 | predator | Observed to occur (Brown 2011) |
| 23 | Syrphus vitripennis | 12 | Predator/pollinator | Observed to occur (Piekarska-Boniecka et al. 2017) |
| 24 | Andrena helvola | 11 | pollinator | Not mentioned as associ. |
| 25 | Oulema melanopus | 11 | herbivore | Not mentioned as associ. |
| 26 | Anotylus nitidulus | 10 | predator | Not mentioned as associ. |
| 27 | Armadillidium nasatum | 10 | detritivore | Not mentioned as associ. |
| 28 | Episyrphus viridaureus | 10 | Predator/pollinator | Not mentioned as associ. |
| 29 | Andrena haemorrhoa | 9 | pollinator | Observed to occur (Chambers 1946) |
| 30 | Ectopsocus californicus | 9 | herbivore | Not mentioned as associ. |
| 31 | Scatopsciara vitripennis | 9 | other | Not mentioned as associ. |
| 32 | Cacopsylla melanoneura | 8 | herbivore | Associ. closely (Tedeschi et al. 2002) |
| 33 | Cylindroiulus punctatus | 8 | detritivore | Not mentioned as associ. |
| 34 | Lepidocyrtus cyaneus | 8 | detritivore | Not mentioned as associ. |
| 35 | Meliscaeva auricollis | 8 | Predator/pollinator | Observed to occur (Stanic 2024) |
| 36 | Amara aenea | 7 | predator | Not mentioned as associ. |
| 37 | Camptocladius stercorarius | 7 | pollinator | Not mentioned as associ. |
| 38 | Cymbaeremaeus cymba | 7 | predator | Not mentioned as associ. |
| 39 | Dryophilocoris flavoquadrimaculatus | 7 | herbivor/predator | Not mentioned as associ. |
| 40 | Micreremus brevipes | 7 | predator | Not mentioned as associ. |
| 41 | Orthosia cerasi | 7 | herbivore/pollinator | Not mentioned as associ. |
| 42 | Oxythyrea funesta | 7 | pollinator | Not mentioned as associ. |
| 43 | Philoscia muscorum | 7 | detritivore | Not mentioned as associ. |
| 44 | Scaptomyza pallida | 7 | pollinator | Not mentioned as associ. |
| 45 | Amischa analis | 6 | predator | Not mentioned as associ. |
| 46 | Anthonomus pomorum | 6 | herbivore | Associ. Closely (Dicker 1946) |
| 47 | Empis chioptera | 6 | predator | Not mentioned as associ. |
| 48 | Frankliniella intonsa | 6 | herbivore | Not mentioned as associ. |
| 49 | Sitona lineatus | 6 | herbivore | Not mentioned as associ. |
| 50 | Tomocerus vulgaris | 6 | detritivore | Not mentioned as associ. |
| **Leaf** |  |  |  |  |
| 1 | Aculus schlechtendali | 86 | herbivore | Associ. closely (Ellis WN 2025) |
| 2 | Apis mellifera | 46 | pollinator | Associ. closely (Verma and Dulta 1986) |
| 3 | Typhlodromus pyri | 26 | predator | Observed to occur (Croft and Macrae 1993) |
| 4 | Operophtera brumata | 24 | herbivore/pollinator | Associ. closely (Hand et al. 1987) |
| 5 | Delia platura | 17 | herbivore/pollinator | Observed to occur (Boucher et al. 2021) |
| 6 | Dysaphis plantaginea | 11 | herbivore | Associ. Closely (Dib et al. 2010) |
| 7 | Melolontha melolontha | 10 | herbivore | Observed to occur (Lakatos and Tóth 2006) |
| 8 | Thrips fuscipennis | 10 | herbivore | Observed to occur (Badowska-Czubik et al. 2006) |
| 9 | Euseius amissibilis | 9 | predator | Not mentioned as associ. |
| 10 | Armadillidium vulgare | 8 | detritivore | Not mentioned as associ. |
| 11 | Melolontha hippocastani | 8 | herbivore | Observed to occur (Kula 2022) |
| 12 | Cacopsylla melanoneura | 6 | herbivore | Associ. closely (Tedeschi et al. 2002) |
| 13 | Ectopsocus californicus | 6 | herbivore | Not mentioned as associ. |
| 14 | Lasius psammophilus | 6 | other | Not mentioned as associ. |
| 15 | Lycoriella sativae | 6 | other | Not mentioned as associ. |
| 16 | Anthonomus pomorum | 5 | herbivore | Associ. Closely (Dicker 1946) |
| 17 | Asymmetrasca decedens | 5 | herbivore | Not mentioned as associ. |
| 18 | Coccinella septempunctata | 5 | predator | Observed to occur (Radwan and Lövei 1982) |
| 19 | Epuraea melanocephala | 5 | herbivore/pollinator | Not mentioned as associ. |
| 20 | Dermanyssus gallinae | 4 | other | Not mentioned as associ. |
| 21 | Magdalis ruficornis | 4 | herbivore | Observed to occur (Grossheim 1930) |
| 22 | Orchesella villosa | 4 | detritivore | Not mentioned as associ. |
| 23 | Porcellionides pruinosus | 4 | detritivore | Not mentioned as associ. |
| 24 | Scathophaga stercoraria | 4 | pollinator | Not mentioned as associ. |
| 25 | Aptinothrips rufus | 3 | herbivore | Not mentioned as associ. |
| 26 | Botanophila fugax | 3 | herbivore/pollinator | Observed to occur (Copoiu and Purcărea 2023) |
| 27 | Bradysia inusitata | 3 | other | Not mentioned as associ. |
| 28 | Chrysoperla carnea | 3 | predator | Observed to occur (Yazdanabad et al. 2022) |
| 29 | Harmonia axyridis | 3 | predator | Observed to occur (Brown 2011) |
| 30 | Helophilus hybridus | 3 | predator/pollinator | Observed to occur (Sekretarczyk and Piekarska‐Boniecka 2012) |
| 31 | Lepidocyrtus cyaneus | 3 | detritivore | Not mentioned as associ. |
| 32 | Orthonychiurus folsomi | 3 | detritivore | Not mentioned as associ. |
| 33 | Scaptomyza pallida | 3 | pollinator | Not mentioned as associ. |
| 34 | Syrphus vitripennis | 3 | pollinator | Observed to occur (Piekarska-Boniecka et al. 2017) |
| 35 | Thrips major | 3 | herbivore | Observed to occur (Pobożniak 2008) |
| 36 | Abacarus lolii | 2 | herbivore | Not mentioned as associ. |
| 37 | Andrena helvola | 2 | pollinator | Not mentioned as associ. |
| 38 | Anotylus nitidulus | 2 | predator | Not mentioned as associ. |
| 39 | Aphodius granarius | 2 | detritivore | Not mentioned as associ. |
| 40 | Balaustium murorum | 2 | herbivore | Not mentioned as associ. |
| 41 | Calliphora vomitoria | 2 | pollinator | Not mentioned as associ. |
| 42 | Cheiracanthium mildei | 2 | predator | Not mentioned as associ. |
| 43 | Cylindroiulus punctatus | 2 | detritivore | Not mentioned as associ. |
| 44 | Drosophila subobscura | 2 | other | Observed to occur (Silva-López et al. 2023) |
| 45 | Elipsocus moebiusi | 2 | other | Not mentioned as associ. |
| 46 | Epistrophe eligans | 2 | predator/pollinator | Observed to occur (Nagy et al. 2015) |
| 47 | Episyrphus viridaureus | 2 | pollinator | Not mentioned as associ. |
| 48 | Limnophyes habilis | 2 | other | Not mentioned as associ. |
| 49 | Lycoriella agraria | 2 | other | Not mentioned as associ. |
| 50 | Lycoriella ingenua | 2 | other | Not mentioned as associ. |

.


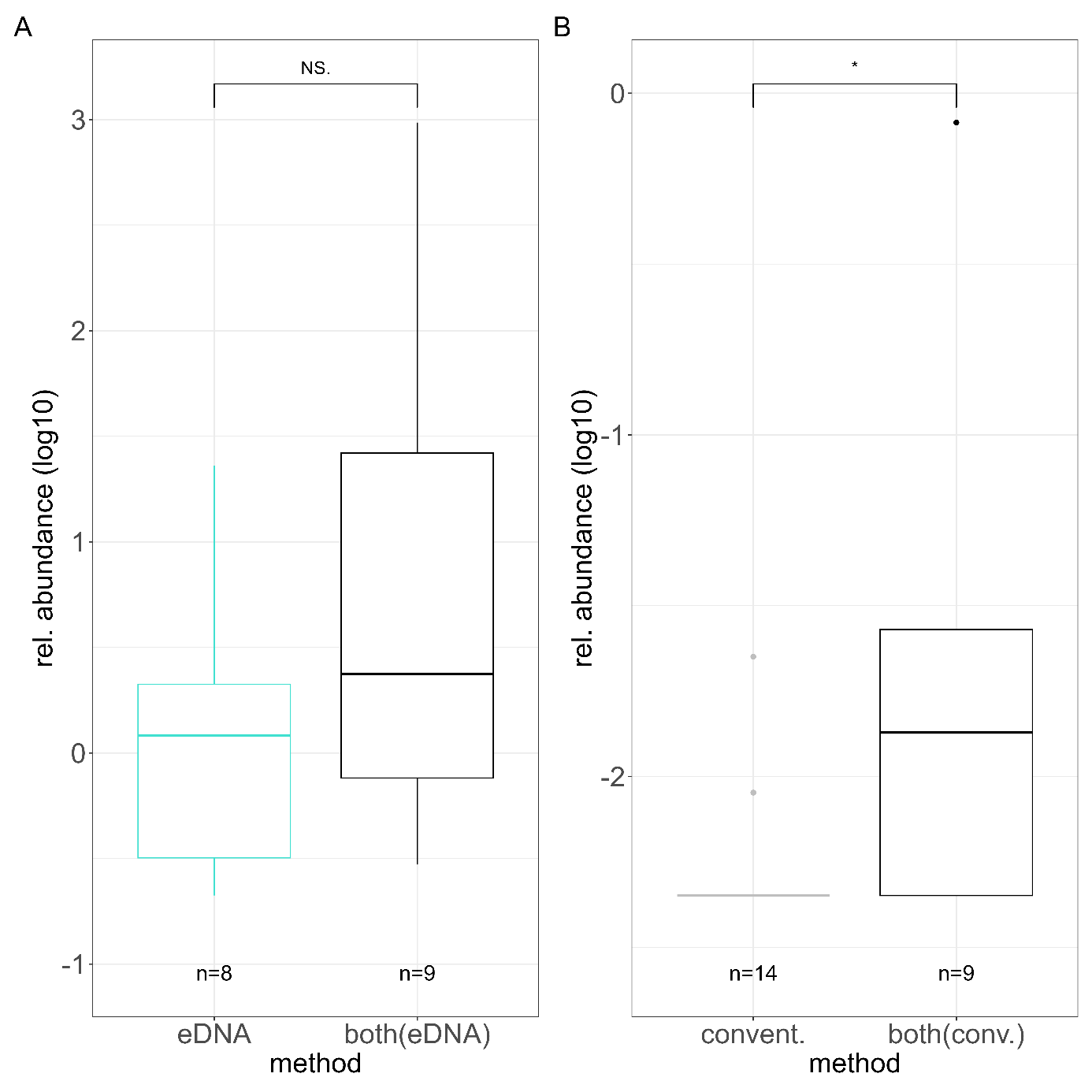


Supplementary figure 1: Comparison of the bee species detected by the conventional method and the eDNA methods (apple leaves and flowers). A) Read abundance of the 5 bee species detected by both methods (eDNA and traps) and the 11 bee species detected exclusively by the eDNA method. B) Relative abundance of the 3 bee species detected exclusively in the traps and the 5 bee species detected by the two methods and the.
